# Lipocalin-2 deficiency may predispose to the progression of spontaneous age-related adiposity in mice

**DOI:** 10.1038/s41598-020-71249-7

**Published:** 2020-09-03

**Authors:** Keya Meyers, María López, Joanna Ho, Savannah Wills, Srujana Rayalam, Shashidharamurthy Taval

**Affiliations:** 1 Department of Pharmaceutical Sciences, School of Pharmacy, Philadelphia College of Osteopathic Medicine – Georgia Campus, 625 Old Peachtree Road, Suwanee, GA 30024 USA; 2 Department of Pharmaceutical Sciences, School of Pharmacy, Philadelphia College of Osteopathic Medicine, Room 3040, 625 Old Peachtree Road, Suwanee, GA 30024 USA; 3 Department of Pharmaceutical Sciences, School of Pharmacy, Philadelphia College of Osteopathic Medicine, Room 3031, 625 Old Peachtree Road, Suwanee, GA 30024 USA

**Keywords:** Inflammation, Obesity

## Abstract

Lipocalin-2 (Lcn2) is an innate immune protein elevated by several orders of magnitude in various inflammatory conditions including aging and obesity. Recent studies have shown that Lcn2 is secreted by adipocytes in response to inflammation and is categorized as a new adipokine cross-linking innate immunity and metabolic disorders including obesity. However, the involvement of Lcn2 and its function during the progression of obesity is largely unknown. Recently, browning of white adipose tissue (WAT) has gained attention as a therapeutic strategy to combat obesity. Herein, we have shown that treatment of mature 3T3-L1 adipocytes with recombinant Lcn2 (rec-Lcn2) resulted in the up-regulation of thermogenic and beige/brown markers (UCP1, PRDM16, ZIC-1 and TBX1) and increased mitochondrial activity. Additionally, global Lcn2 genetic knockout (Lcn2KO) mice exhibited accelerated weight gain and visceral fat deposition with age, when compared to wild type (WT) mice. Taken together, both in vitro and in vivo studies suggest that Lcn2 is a naturally occurring adipokine, and may serve as an anti-obesity agent by upregulating the thermogenic markers resulting in the browning of WAT. Therefore, Lcn2 and its downstream signaling pathways could be a potential therapeutic target for obesity.

## Introduction

Obesity is a chronic low-grade inflammatory disorder. With nearly 40% of the adult population in the United States being obese, it is no longer just a problem of uncontrolled weight gain but considered as a chronic disease of epidemic proportions. Obesity increases the risk of several disorders, including type-2 diabetes, cardiovascular disease, stroke and bone loss^1^. The expense of treating obesity-related illnesses is also increasing and now accounts for nearly 28% of total health-care costs^2^. It is estimated that obesity treatment costs between $147 and 210 billion per year, with another $4.3 billion in lost productivity^3–5^. Despite the global burden of the disease, there are limited FDA approved anti-obesity drugs available and their use is tempered by significant adverse side effects^6–8^. As obesity is now a national epidemic, there is a critical need for the development of anti-obesity drugs with fewer side effects. Adipose tissue plays a vital role in regulating the metabolic disorders and inflammation. White adipose tissue (WAT) primarily functions as an energy storage unit and is a source for inflammatory mediators^9–12^. Dysfunction of WAT leads to progression of obesity and its related metabolic disorders^13^. Recent studies have shown that conversion of WAT to brown-like beige adipose tissue, known as browning of WAT, is a promising target for the treatment of obesity and associated metabolic and inflammatory disorders^13^. Browning of WAT, accompanied by increased mitochondrial activity and thermogenesis, has been reported to decrease weight gain in the high fat diet (HFD) fed obese mouse models^14^.

More recently, lipocalin 2 (Lcn2) is categorized as a new adipokine secreted by various cell types including adipocytes. It has also been reported that Lcn2 is upregulated systemically by several fold during multiple pathological conditions^15–18^. Further, others and we have shown that Lcn2 is indispensable for neutrophil recruitment, adhesion and function during various inflammatory conditions in animal models and may act as an anti-inflammatory agent to promote tissue regeneration^19–21^. However, its physiological role in metabolic disorders is still obscure. Studies have shown that Lcn2 is upregulated in adipose tissue and liver of ob/ob mice suffering from severe hyperglycemia and hyperinsulinemia when compared with their lean littermates^22,23^. Elevated glucose levels induced Lcn2 expression and insulin treatment restored glucose levels reducing Lcn2 levels in vivo^24,25^. In support of this, treating the mature adipocytes with insulin when cultured in high glucose media markedly increased Lcn2 levels while the insulin effect was lost when cultured in low glucose media^26^ confirming the regulation of Lcn2 levels by blood glucose. Upregulation of Lcn2 was also reported in HFD fed rodent models and obese people^27,28^. Additional studies support diabetic and obesogenic effects of Lcn2 where, Lcn2 deficiency attenuated insulin resistance and the percentage body weight gain and blood glucose levels were lower compared to WT mice^29^. More recently, Ishii et al. reported anti-thermogenic and obesity-promoting effects of Lcn2 in HFD fed mice^30^. On the contrary, several studies showed that Lcn2KO mice gained more weight, developed dyslipidemia and insulin resistance with HFD compared to their WT littermates^31–35^.

It is possible that Lcn2 probably acts primarily as an anti-inflammatory agent in the adipocytes. Since diabetes, obesity and metabolic syndrome are all associated with underlying inflammation, Lcn2 treatment may potentially address several metabolic conditions. In fact, exogenous Lcn2 attenuated tumor necrosis factor (TNF) mediated expression of PPARγ and GLUT4 in adipocytes^36^ and reduced TNF levels in the adipose tissue significantly mitigating inflammation in Lcn2KO mice^29^. These reports suggest that Lcn2 has a paradoxical role in metabolic disorders. The conflicting reports on the effects of Lcn2 on obesity warrant additional studies to investigate the mechanism behind the pro/anti-obesity effect of Lcn2. In this report, using Lcn2KO and WT mice as well as in vitro studies with recombinant Lcn2 (rec-Lcn2), we have investigated the anti-obesity functions of Lcn2. We observed that around 40% of female Lcn2KO mice but not male mice developed obesity with age as evidenced by weight gain and adiposity. Further, treatment of mature 3T3-L1 adipocytes with rec-Lcn2 upregulated the key browning/beiging markers (TBX1, ZIC1, UCP1 and PPARγ) and mitochondrial content. Our data support the anti-obesity effects of Lcn2 and thus Lcn2 deficiency may predispose to age-related development of metabolic disorders.

## Results

### The Lcn2 receptor (24p3R) is expressed on both undifferentiated (pre) and differentiated (mature) adipocytes

**Figure 1:**
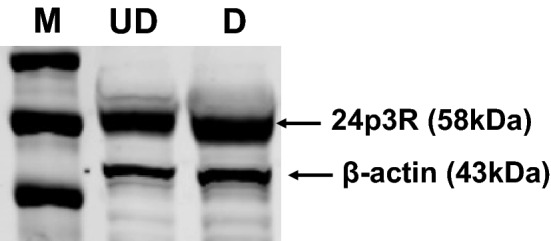
Lcn2 receptor (24p3R) is expressed in 3T3-L1 adipocytes. 3T3-L1 pre-adipocytes were cultured and differentiated to mature adipocytes as described under “Materials and methods” section. The Lcn2 receptor (24p3R) expression in 3T3-L1 cells was confirmed by western blot analysis. *M* markers, *UD* undifferentiated cells, *D* differentiated cells. Data shown are representative of two individual experiments. The picture represents the cropped blot and the uncropped image is provided in Supplementary Fig. S1.

We investigated the expression of Lcn2 receptor in murine pre- and mature 3T3-L1 adipocytes by western blot analysis. 3T3-L1 cells were differentiated to mature adipocytes as described previously^37^. We observed that both pre- and mature adipocytes express 24p3R protein (Fig. 1). These results suggest that adipocytes are capable of transporting the soluble Lcn2 in and out of the cells. It has been shown that 24p3R protein is expressed in murine tissues including heart, lung, liver, spleen, kidney, stomach, small intestine, skeletal muscle, ovary, thymus, testis, uterus and placenta^38^. Langelueddecke et al.^39^ reported that 24p3R is not expressed in WKPT-0293 Cl (rodent proximal tubule), HeLa (human cervical cancer), Jurkat (human T lymphocyte) and D1-F4 (murine immature T cell) cell lines. Further, Megalin/LRP2 has been identified in several studies as a key receptor in facilitating up-stream effects of Lcn2^40^. In the current study, while we demonstrate the expression of 24p3R in 3T3-L1 adipocytes for the first time, we report that the effects of Lcn2 in 3T3-L1 cells are mediated partly through 24p3R and it is possible that LRP2 or other unknown molecules may also play a role in facilitating the effects of Lcn2.

### Exogenous supply of recombinant Lcn2 (rec-Lcn2) is not toxic to 3T3-L1 cells at the tested doses

**Figure 2:**
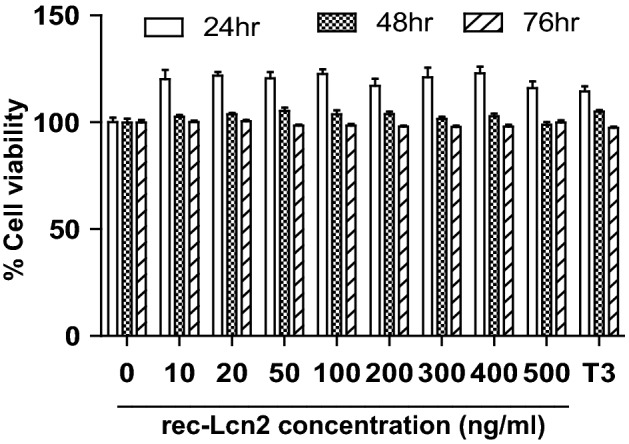
rec-Lcn2 and T3 did not alter the viability of 3T3-L1 cells at the tested dose. Mature 3T3-L1 cells were treated with different concentration of rec-Lcn2 and T3 separately for 24–76 h. The cell viability was estimated using Prestoblue Cell Viability Reagent according to manufacturer’s protocol. Absorbance was recorded at 415 nm using an HT Synergy microplate reader. Values for percent cell viability were normalized to the cell viability of cells with no exposure to rec-Lcn2 (vehicle control). Data shown are the average of two individual experiments. Each experiment was carried in triplicate. P value < 0.05 considered as statistically significant. Two-way ANOVA with Bonferroni post-Hoc test was used to calculate the statistical significance.

To investigate the cytotoxicity effect of rec-Lcn2, 3T3-L1 mature adipocytes were treated with rec-Lcn2 (0–500 ng/ml) for 24–76 h. The cell viability was measured using PrestoBlue cell viability kit. Lcn2 did not decrease cell viability of 3T3-L1 mature adipocytes at as high as 500 ng/ml of culture supernatant (Fig. 2). The physiological concentration of Lcn2 in serum is 100 ng/ml^41^. These results suggest that the Lcn2 is a natural molecule and may not exert the toxic effect when physiologically higher concentration of Lcn2 is being used to treat cells.

### rec-Lcn2 upregulates brown/beige and thermogenic markers in mature 3T3-L1 adipocytes

**Figure 3:**
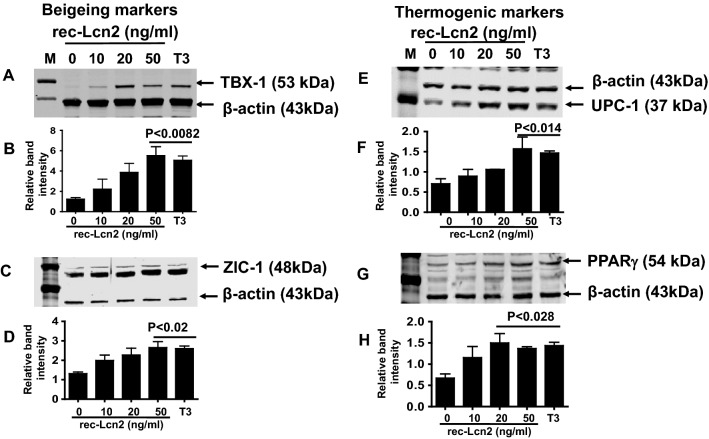
Exogenous rec-Lcn2 upregulates beigeing and thermogenic markers in mature adipocytes (3T3-L1). 3T3-L1 cells were cultured and treated with various concentrations of rec- Lcn2 (0–50 ng/ml). After 24hrs, the cells were harvested, lysed with RIPA buffer and western blot was carried out to detect the beiging (**A**,**C**) and thermogenic markers (**E**,**G**). *M* markers, *T3* Triiodothyronine. The picture represents the cropped blots from individual gels and the uncropped images are provided in Supplementary Fig. S3A,C,E,G. Data shown are the representative of three individual experiments. Relative band intensity (**B**,**D**,**F**,**H**) was calculated as described under “Materials and methods” section. One-way ANOVA with Newmann-Keuls post-hoc test was used to calculate the statistical significance.

3T3-L1 mature adipocytes were treated with different concentrations of rec-Lcn2 (0–50 ng/ml). Western blot analysis was carried out to detect the expression of beige/brown and thermogenic markers. Triiodothyronine (T3) hormone was used as a positive control. Protein levels of beige/brown markers TBX1, ZIC1, UCP-1 and PPARγ were upregulated by rec-Lcn2 treatment in a dose-dependent fashion (Fig. 3A,C,E,G). Statistical analysis suggested that 50 ng/ml of rec-Lcn2 was enough to upregulate these markers (Fig. 3B,D,F,H). Further, additional thermogenic markers such as PGC-1α and PRDM16 were also upregulated in response to rec-Lcn2 (data not shown). These results suggest that supplementation with exogenous Lcn2 upregulates thermogenic markers in adipocytes.

### rec-Lcn2 increases mitochondrial mass in 3T3-L1 adipocytes

**Figure 4:**
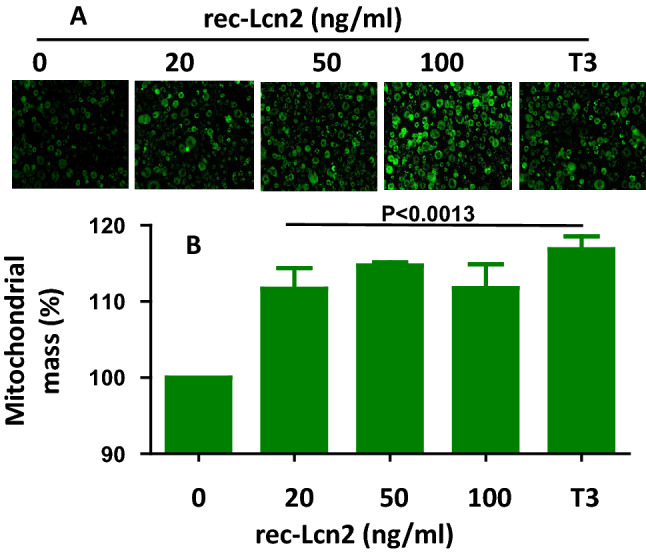
rec-Lcn2 increases mitochondrial mass. Mature 3T3-L1 cells were cultured and treated with various concentration of rec-Lcn2 (0–50 ng/ml). After 24hrs, the cells were treated with MitoTracker reagents as described under material and methods. Photographs (**A**) and data (**B**) are representative of two individual experiments; experiment was carried out in triplicate. P value < 0.05 considered as statistically significant. One-way ANOVA with Newmann-Keuls post-hoc test used to calculate the statistical significance.

We next investigated whether the increase in thermogenic marker expression by exogenous Lcn2 is associated with an increase in the mitochondrial mass in mature white adipocytes. Mature 3T3-L1 cells were treated with rec-Lcn2 (0–100 ng/ml) for 24 h at 37 °C. Mitochondrial mass was detected using green fluorescent Mito/Tracker assay kit (R&D system). T3 was used as a positive control. Cells were photographed with an EOVS-FL AUTO inverted fluorescence microscope and total fluorescence measured with a microplate reader at 516 nm. Mature 3T3-L1 cells treated with rec-Lcn2 (100 ng/ml) exhibited brighter fluorescence than untreated cells (Fig. 4A), suggesting an increased mass of mitochondria within the cells. Quantitation of fluorescence suggests that rec-Lcn2 increased mitochondrial mass by up to 20% (Fig. 4B). These results suggest that exogenous supplementation of Lcn2 increases mitochondrial biogenesis.

### Lcn2 deficient female mice gained more weight and displayed increased visceral fat deposition as they age compared to WT mice

**Figure 5:**
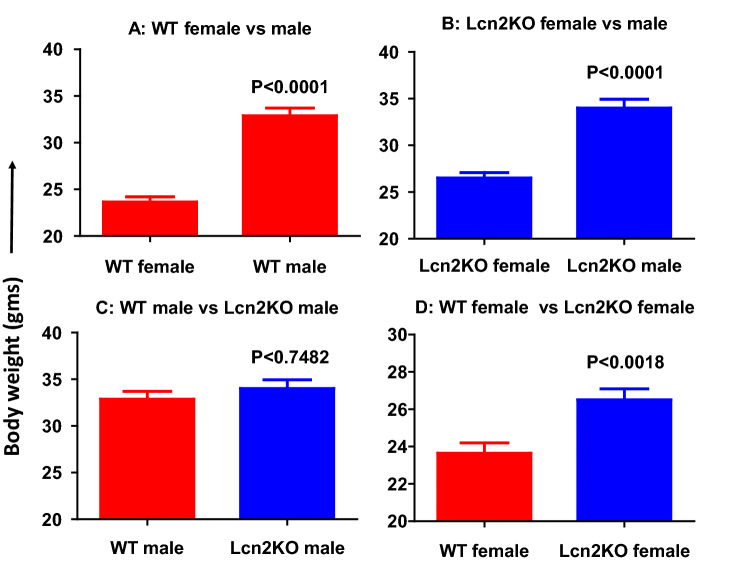
Lcn2 deficiency leads to development of age-related weight gain. Male WT (n = 18), female WT (n = 23), male Lcn2 KO (n = 24) and female Lcn2KO (n = 20) mice were fed on regular chow diet for 1 year. Average body weight of WT male and female (**A**), and Lcn2KO male and female (**B**) mice. Average body weight of WT versus Lcn2KO male (**C**) and female (**D**). P value < 0.05 considered as significant. Nonparametric Two-tailed t-test (Mann–Witney) was performed to compare the statistical significance.

**Figure 6:**
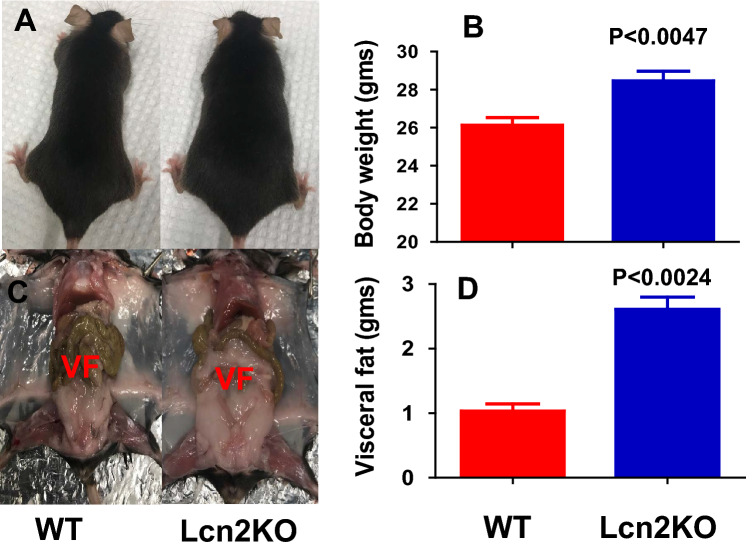
Lcn2 deficient female mice exhibited age-related weight gain and visceral fat deposition. One-year old female Lcn2 KO mice and WT mice weighing more than 25 g mice on regular chow diet. (**A**,**C**) Representative photographs of Lcn2KO and WT mice with weight gain and visceral fat (VF) accumulation. (**B**) Average of body weight of Lcn2KO (n = 8) and WT (n = 6) mice. (**D**) Average weight of visceral fat from Lcn2KO and WT mice. P value < 0.05 considered as significant. Nonparametric Two-tailed t-test (Mann-Witney) were performed to compare the statistical significance.

It is unclear whether Lcn2KO mice are susceptible or resistant to developing obesity^29–35^. Thus far, there are no studies investigating the role of Lcn2 on age-dependent weight gain or fat deposition. In this study, we investigated the weight gain in 1-year-old sex and age matched WT male (n = 15), WT female (n = 20), Lcn2KO male (n = 21) and Lcn2KO female (n = 17) mice under normal chow diet. As expected, there is significant increase in total body weight of male compared to female mice in both WT and Lcn2KO mice (Fig. 5A,B). Interestingly, we observed increased weight gain in female Lcn2KO mice (Fig. 5D). However, we did not observe significant body weight gain in Lcn2KO male mice (Fig. 5C) compared to WT mice. For further analysis, we considered the male mice weighing above 35gm (~ 60% mice in this group are between 30 and 35 g) and female mice more than 25gm (~ 60% are between 20 and 25 g) as overweight mice (Table 1). We selected female Lcn2KO and WT mice as they exhibited significant difference in weight than male mice, in addition, it has been shown that age-dependent weight gain is more significant in women than men^42^. Interestingly, we observed that only 40–45% (8 out of 17) of the one-year-old Lcn2KO mice gained significantly more weight compared to WT mice (age and sex matched) fed on a regular chow diet (Fig. 6A,B). However, there was a significant increase in the weight gain in female Lcn2KO mice than WT when the overall population of the mice was considered (Fig. 5D). In addition, we also observed an increase in visceral adipose tissue deposition in Lcn2KO mice (Fig. 6C,D). These observations along with our in vitro studies with rec-Lcn2 (Fig. 3) suggest that Lcn2 insufficiency may lead to the spontaneous development of obesity mediated partly through the inability to induce browning of WAT.

**Table 1:** Weight in grams of the WT and Lcn2KO mice.

WT		Lcn2 KO	
Male (n = 15)	Female (n = 20)	Male (n = 21)	Female (n = 17)
33.2	20.6	*39.1*	*30.2*
32.4	22.1	30.5	*26.2*
30.2	25.4	33.4	*30.5*
*35.7*	23.4	32.1	*27.5*
32.8	24.7	33.1	25.4
34.3	22.7	23.6	22
*34.6*	18.4	29	*28*
33.6	*25.7*	33.5	26.5
32.5	19.2	*38.2*	26.6
*35.5*	24.2	30.6	22.5
30.2	*27.8*	*39.9*	23.5
23.6	24.8	33.5	*28.5*
*35.1*	*25.6*	33	25
*36.9*	*25.5*	32.3	*28.9*
32.6	*26.8*	*40.1*	25.6
	22.4	33.3	*27.9*
	21.2	32.9	25.7
	24.4	*42.5*	
	*25.4*	32	
	22.6	33.6	
		*37.8*	

The italicized weight of the mice considered as over-weight mice as described in the text.

### Systemic Lcn2 upregulation is age-dependent

**Figure 7:**
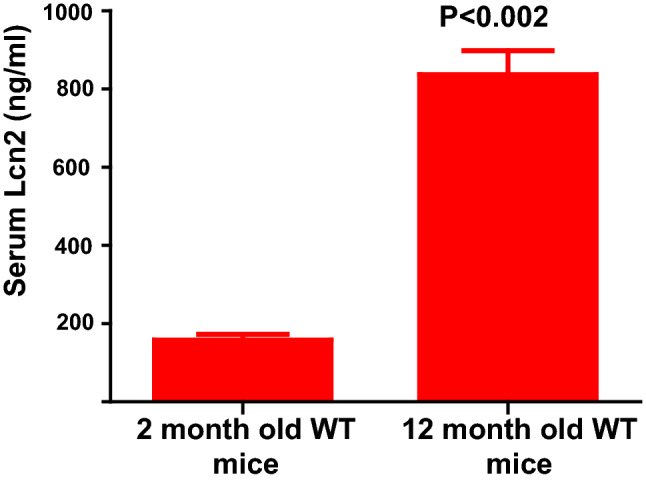
Systemic levels of Lcn2 is upregulated in WT mice as they age. Serum samples were collected from 2-month (n = 4) and 1-year-old (n = 10) WT mice. ELISA was carried out to detect the Lcn2 concentration in WT mice serum and quantified using the standard as per the manufacturer’s protocol. Experiment was carried out in triplicate. P value < 0.05 considered as significant.

Fat accumulation increases with age in humans and mice. To study whether systemic Lcn2 levels increase with age in WT mice, we collected serum samples from 2- (n = 4) and 12-month old (n = 10) WT mice and quantified the systemic upregulation of Lcn2 by ELISA (R&D system). Lcn2 was more than five-fold higher in 12-month than 2-month old mice (Fig. 7). We observed the consistency between the increase in serum levels at 12 months and the weight gain of 12-month-old Lcn2 deficient mice compared to WT mice (Fig. 5). However, the upregulation of Lcn2 may have implications other than a protective response towards obesity. Owing to our in vitro and in vivo observations (Figs. 3, 4, 5, 7), we partly attribute this characteristic increase in serum Lcn2 levels with age as a defense mechanism to help modulate developing adiposity.

## Discussion

Recently, we and others have shown the protective function of Lcn2 during acute and chronic inflammatory^21,43,44^ and metabolic disorders in mice^33,45–51^. Several reports suggest the conflicting role of Lcn2 as a pro and anti-inflammatory agent. The increase in systemic levels of Lcn2 and its effects have been explored by previous studies with the purpose of identifying the immune-mediating role in specific inflammatory disorders. In our previous study, we have shown that, under acute skin inflammation model, Lcn2 is required to attract the inflammatory cells at the site of inflammation suggesting the pro-inflammatory effect. However, during chronic conditions such as autoimmune arthritis, Lcn2 deficiency exacerbate the arthritis suggesting its beneficial role during chronic inflammatory conditions^21^. Further, rec-Lcn2 protected the development of nephritis in an animal model^52^. In addition, we also observed that supplementing the Lcn2 polarizes the macrophages towards the alternative (M2) phenotype (unpublished data). Taken together, these studies suggests that Lcn2 may have differential function depending on the particular pathological conditions.

Although Lcn2 is known to be upregulated during various inflammatory conditions, its biological activity during the progression of chronic inflammatory metabolic disorders such as obesity is still controversial. In this report, we have shown that the rec-Lcn2 supplementation potentiates the upregulation of key thermogenic markers and of mitochondrial content in 3T3-L1 adipocytes. In addition, we have observed only female but not male Lcn2KO mice gained weight as they reach one-year of age compared to WT mice. Gao et al.^31^ reported that 30 week old Lcn2KO mice gained weight, whereas Ishii et al.^30^ showed no difference in body weight between 28 week old Lcn2KO and WT mice with regular chow diet. However, Lcn2KO mice showed decreased weight gain and attenuated insulin resistance when fed high-fat diet. We observed that only 40–45% of the female Lcn2KO mice gained weight as they age (1 year old) than WT mice on regular chow diet. The reason for ~ 60% Lcn2KO mice not gaining weight in our study is unclear. However, our data is consistent with the previous reports^31–35^ that the lack of Lcn2 predisposes the mice to progression of weight gain through impaired thermogenesis and possibly browning of WAT. The reason for no significant difference in male Lcn2KO mice compared to WT mice is not clear. However, this could be due to the absence of female sex hormone estrogen as it has been shown that female Lcn2KO mice have altered estradiol secretion and its receptor signaling^53^.

Lcn2 deficiency was shown to decrease serum estrogen levels and estrogen receptor alpha (ERα) activity. Since estrogens are major regulators of lipid metabolism, Lcn2 deficiency may lead to metabolic dysregulation leading to an increase in weight gain^53^. Our observations on sex-specific effects of Lcn2 are in line with the findings of Guo et al.^53^. On the contrary, Chella et al.^54^ demonstrated that Lcn2 downregulated ERα expression in female adipose tissue and suggest that the female-specific negative regulation by Lcn2 could be due to an intricate feedback mechanism^54^. However, others have shown that when ovariectomized mice were treated with 17β-estradiol, an increase in serum Lcn2 level was observed suggesting a positive correlation between estrogen and Lcn2^55^. More recent studies with adipose tissue from postmenopausal women also report a dose-dependent effect of 17-β-estradiol on Lcn2 expression suggesting a positive correlation^56^. Interestingly, Kamble et al. report that estrogen-driven Lcn2 effects are mediated through ERβ and that both ERα and ERβ pathways have opposite effects on Lcn2 expression. These observations suggest that additional studies are warranted to understand the sex-dependent roles of Lcn2.

While we hypothesize that exogenous administration of rec-Lcn2 may have anti-obesity effects in the Lcn2KO mice, the significance of increased systemic Lcn2 levels with age (Fig. 7) in the context of obesity is not clearly understood. Previous reports suggest elevated levels of Lcn2 in obesity as a protective mechanism against inflammation and insulin resistance^31^. Since obesity is associated with chronic low-grade inflammation, increase in visceral adiposity and weight gain seen in Lcn2KO mice may be partly due to the absence of Lcn2-mediated anti-inflammatory effects. Additionally, it is possible that the elevated levels of Lcn2 in adipose tissue microenvironment has specific browning effects but the elevation of systemic Lcn2 levels may have other possible implications in addition to being a protective response towards an increase in adiposity. Further studies are needed to elucidate the relationship between the elevated systemic Lcn2 levels and changes in adiposity and/or browning of WAT.

Lcn2 was recently reported to regulate the activation of BAT^32^. The unique ability of BAT to dissipate energy stored in the triglycerides as heat, makes it an attractive target for anti-obesity therapy. Furthermore, brown adipocytes have the intrinsic ability to dampen the inflammatory response, as opposed to the pro-inflammatory effects of white adipocytes^57^. It was believed that adult humans lose functional BAT with age. However, in the past decade several reports confirmed the existence of metabolically active, brown-like adipose tissue called beige adipose tissue (BeAT) in adult humans^58^. Expansion or activation of BeAT is associated with a healthy metabolic profile and reduction of fat mass^59,60^. Additionally, conversion of WAT to BAT, referred to as browning of WAT, confers protection against diet-induced obesity in rodents^14^. In this study, we have investigated for the first time the potential anti-obesity effects of rec-Lcn2 mediated through the browning of WAT.

BAT has the ability to convert chemical energy into heat through uncoupling protein 1 (UCP1), which promotes mitochondrial biogenesis and uncouples oxidative phosphorylation to generate heat^61–63^. UCP1 is activated by the peroxisome proliferator-activated receptor gamma coactivator (PGC) 1-α whose transcription is regulated by p38 mitogen-activated protein kinase (p38MAPK)^64^. Deis et al. reported that Lcn2 deficiency decreases the expression of UCP1 and PGC1α in inguinal WAT in mice^65^. Furthermore, adipocytes isolated from Lcn2KO mice have attenuated p38 mitogen-activated protein kinase (p38MAPK) signaling pathway activation^32,65^. In this study, we have demonstrated the upregulation of thermogenic markers in response to rec-Lcn2 treatment in an in vitro model. It is possible that Lcn2 has direct effect on adipocytes increasing thermogenic markers, but this could also be a secondary effect in concert with other important metabolic pathways. Although we have not observed any changes in the food intake between WT and KO mice, recent reports by Mosialou et al. showed that Lcn2 can cross the blood–brain barrier and may directly affect hypothalamic neurons^66^. Additionally, Ishii et al.^30^ have reported anti-thermogenic effects of Lcn2 in high-fat diet mouse model. The contrasting reports between Ishii et al. and ours towards the effects of Lcn2 on thermogenic markers can be attributed to the differences in the experimental model and the age of mice used. Our in vitro studies are in agreement with Deis et al.^65^ suggesting thermogenic properties of Lcn2 are accompanied by an upregulation of UCP1 and increased mitochondrial content. Additionally, rec-Lcn2 treatment also upregulated the expression of TBX1, a beige specific marker. TBX1, CD137 and transmembrane protein 26 (TMEM26) are beige specific markers^67^ and in this study, we demonstrate for the first time the upregulation of TBX1 with rec-Lcn2 in mature 3T3-L1 adipocytes (Fig. 3A), suggesting a potential role of Lcn2 in browning of WAT.

PPARγ is considered a master regulator of adipogenesis and plays a major role in the process of adipocyte differentiation, lipid storage and metabolism. Additionally, PPARγ is also a key regulating factor in the activation of brown/beige adipocytes leading to an increase in mitochondrial biogenesis and increased energy expenditure. Under varying pathophysiological conditions, PPARγ was shown to bind to distinct transcription factors to exert its multiple functions in the adipose tissue including adipogenesis and acquisition of brown/beige adipocyte identity^68^. Our findings on the expression of PPARγ in mature adipocytes in response to rec-Lcn2 treatment are not in parallel with the previous reports^35,56^. Distinct results in the effects of PPARγ in these studies can be attributed to many variables including different dose of Lcn2, treatment duration and more specifically different experimental conditions. Kamble et al.^56^ used human adipose tissue explants while Jin et al.^35^ reported Lcn2 mediated effects on PPARγ expression in vivo in mice. Additionally, adipocytes at various stages of their life cycle may respond differently to exogenous stimuli^69^. It is possible that the suppressive effect of Lcn2 on PPARγ expression is seen when preadipocytes are treated with Lcn2 during adipogenesis, while the treatment of mature adipocytes may increase PPARγ which in turn recruits PRDM16 leading to the induction of browning. Nevertheless, additional studies are needed to understand the effects of Lcn2 on various stages of the adipocyte life cycle and the transdifferentiation of white to brown-like beige adipocytes.

**Figure 8:**
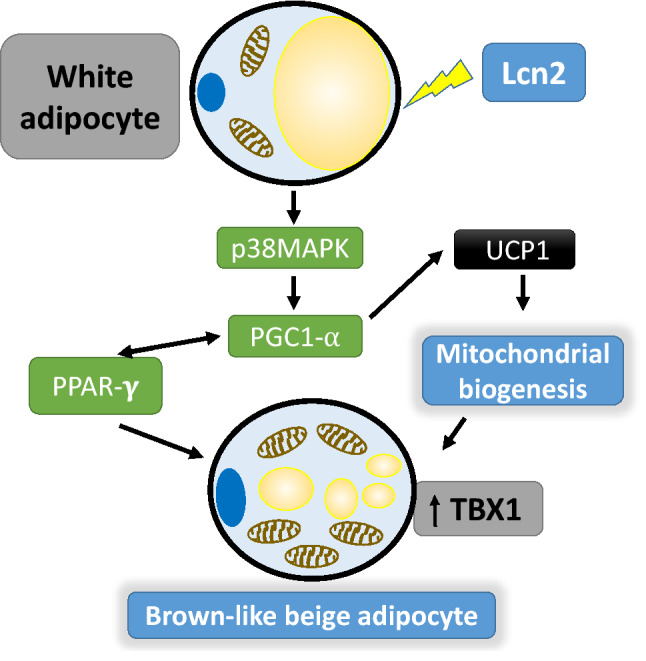
Hypothetical model of Lcn2 mediated transdifferentiation of WAT to BeAT. Lcn2 activates p38MAPK in WAT, which in turn activates PGC1α. The activated PGC1α upregulates UCP1 resulting in mitochondrial biogenesis. PGC1α also activates PPARγ and vice versa. All these events combined with mitochondrial biogenesis converts WAT to BeAT. Brown like beige adipocytes show an upregulation of thermogenic/beige markers, increased mitochondrial content and have smaller lipid droplets.

An increase in PPARγ induces brown-like phenotype in white adipocytes coupled with increased mitochondrial biogenesis and enhanced expression of TBX1^67^. Though administration of PPARγ agonist, rosiglitazone, reduced Lcn2 expression in obese Zucker rats, supplementation of rec-Lcn2 increased the mRNA levels of PPARγ in 3T3-L1 adipocytes^36^. Rosiglitazone induces UCP1 expression to more than double the level induced by beta adrenergic receptor agonists but the mechanisms involving the control of UCP1 expression with exogenous PPARγ agonists are not clearly understood^70^. Nevertheless, we have seen a significant dose-dependent effect of rec-Lcn2 on the expression of PPARγ in mature adipocytes and our results are in agreement with previous findings. Lcn2 deficiency significantly attenuated UCP1 expression in inguinal WAT and primary adipocytes isolated from these tissues showed decreased expression of thermogenic genes, UCP1 and PGC1α^65^. Correspondingly, our in vitro studies also demonstrate a significant increase in TBX1 expression accompanied by an increase in mitochondrial content. Thus, Lcn2 deficiency may contribute to the increase in adiposity coupled with decreased browning of WAT. A hypothetical model of Lcn2-mediated induction of browning in white adipocytes is depicted in Fig. 8.

Our in vitro results on mitochondrial biogenesis are in parallel with Zhang et al.^32^ where mice lacking Lcn2 displayed impaired mitochondrial function and BAT activation in response to cold stimulation and in HFD-induced obesity. However, anti-thermogenic role of Lcn2 has been reported by Chella et al.^54^ using a mouse model with conditional overexpression of Lcn2 in the adipose tissue and liver. The conflicting reports on the effects of Lcn2 on mitochondrial function could be attributed to the fundamental differences in the models used in these studies. Interestingly, Lcn2 overexpression in WAT promoted metabolic disturbances in females but not in males and suggest autocrine/paracrine effects of Lcn2 for mitochondrial dysfunction and inflammation^54^. In contrast, Zhang et al. reported upregulation of PPARγ expression in adipocytes in the presence of rec-Lcn2 and as PPARγ suppresses NF-kB transcriptional activity, suggesting anti-inflammation as the primary role of Lcn2 in adipose tissue^36^.

In conclusion, our data demonstrated that exogenous Lcn2 supplementation induces browning of adipocytes in vitro and is supported by the in vivo evidence of an increase in adiposity and body weight associated with Lcn2 deficiency. Naturally occurring elevation in Lcn2 levels seen in obese mice may not be sufficient to control or reverse the pathological changes induced by obesity. Nonetheless, exogenous supplementation of Lcn2 may confer protection against ongoing obesity and inflammation.

## Material and methods

### Reagents

HRP-substrate and SDS-PAGE gels were from BioRad (Hercules, CA). Iron-, siderophore- and endotoxin-free mouse recombinant Lcn2 (rec-Lcn2) and mouse Lcn2 Duoset ELISA kit were from R&D Systems (Minneapolis, MN), Micro BCA protein assay kit from Pierce (Rockford, IL). The rec-Lcn2 (5 ug/100ul) was supplied in MES buffer with NaCl from the manufacturer; we have added 900 ul of PBS to make it 5 ug/ml. We used 0–50 ng/ml in media, leading to a 1:1,000 dilution. PBS was used as a vehicle control. Cell culture reagents, MitoTracker Green, RIPA lysis buffer, protease/phosphatase inhibitors, Prestoblue Cell Viability Reagent, Pierce BCA protein assay kit and anti-SLC22A17 (24p3R; PA5-20543) were from ThermoFisher Scientific (Waltham, MA, USA). Affinity-purified polyclonal LiCOR secondary antibodies were from LiCOR Odyssey CXL imaging system software (Lincoln, NE, USA). Anti-ß-Actin (ab8226), anti-TBX1 (T-box protein-1: ab109313) anti-ZIC-1 (zinc finger protein-1: ab229262), anti-UCP-1 (uncoupling protein-1: ab10983), Anti-GAPDH (glyceraldehyde 3-phosphate dehydrogenase: ab8245) and anti-α-Tubulin (ab44928) were from Abcam (Cambridge, MA, USA). Anti-PPAR-γ (peroxisome proliferator-activated receptor-gamma: sc-7196) were from Santa Cruz Biotechnology Inc. Anti- PGC1-α (PPARγc1α: NBP1-04676SS) antibody and anti-PRDM16 (PR domain zinc finger protein 16: NBP1-77096) were purchased from Novus Biologicals (Littleton, CO, USA).

### Animal models and cell lines

Murine embryo 3T3-L1 pre-adipocyte cell line was purchased from America Type Culture Collection (Manassas, VA USA). Lcn2 knock-out (Lcn2KO) mice and wild type (WT) C57BL/6 mice were purchased from Jackson laboratory and bred, and maintained at the PCOM animal facility in Suwanee, GA. WT and Lcn2KO mice were fed with regular chow diet. All animal studies were approved by Philadelphia College of Osteopathic Medicine, Institutional Animal Care and Use Committee (PCOM IACUC) and carried out as per PCOM IACUC guidelines.

### Differentiation of 3T3-L1 cells

3T3-L1 preadipocytes are murine embryonic fibroblasts committed towards adipocyte lineage and the cell line is an established model to study adipocyte biochemistry. 3T3-L1 cells at passage numbers between 6 and 15 were used and the differentiation potential of the cells was determined by the morphological changes and lipid deposition after the induction of differentiation process where preadipocytes develop into mature adipocytes. Differentiation of 3T3-L1 preadipocytes was carried out as we previously described^71,72^. Briefly, 3T3-L1 preadipocytes were cultured in medium containing Dulbecco’s Modified Eagle Medium (DMEM) GlutaMAX with 10% calf serum and 1% penicillin–streptomycin. 3T3-L1 preadipocytes were allowed to grow to appropriate confluency and induced to differentiate using differentiation media I (DM-I) containing DMEM/F12 GlutaMAX with 10% fetal bovine serum, 1% PS, 5 μM dexamethasone (DEXA), 0.5 μg/ml insulin, 0.5 mM 3-isobutyl-1-methylxanthine, 1 μM rosiglitazone and differentiation media II (DM-II) containing DMEM/F12 GlutaMAX with 10% FBS, 1% PS, 0.5 μg/ml insulin and 1 μM dexamethasone (DEXA). For differentiation, post-confluent 3T3-L1 preadipocytes were cultured in DM-I for 3 days followed by DM-II for 4 days to complete the process of adipogenesis and convert preadipocytes into fully differentiated mature adipocytes. Preadipocytes before the induction of differentiation were considered as undifferentiated cells (UD) and mature cells at Day 7 after the induction of differentiation are considered differentiated cells (D).

### Cell viability assay

Mature adipocytes were cultured in 96-well plate and treated with various concentration of rec-Lcn2 (0–500 µg/ml) and T3 for 24–76 h. Cell viability was measured after treatment using Prestoblue Cell Viability Reagent according to manufacturer’s protocol. Absorbance of metabolically active cells was measured 1 h after adding the reagent to the rec-Lcn2 treated and untreated cells using an HT Synergy microplate reader at 570 nm. Cell viability was calculated using the formula % cell viability = OD of individual well/average of control wells OD × 100 (e.g. 0.696/0.721 × 100 = 100.069%).

### Estimation of mitochondrial biogenesis

Mature adipocytes were treated with various concentration of rec-Lcn2 (0–100 ug/ml) for 24-h and incubated with MitoTracker Green according to manufacturer’s protocol. Fluorescent intensity of mitochondrial content was measured using the HT Synergy microplate reader at 516 nm.

### Western blot analysis

Mature 3T3-L1 adipocytes were treated with various concentrations (0–50 ug/ml of culture supernatant) of rec-Lcn2 for 24-h. Cells were washed and suspended with ice-cold RIPA lysis buffer with protease and phosphatase inhibitors. Whole cell lysate was prepared by centrifuging for 10 min at 13,300×*g* at 4 °C. Protein concentrations were determined using the Pierce BCA Protein assay kit. Proteins were separated using 10% SDS polyacrylamide gels and transferred onto Immunoblot Plovinylidene fluoride membranes with the Trans Blot Turbo system (Bio-Rad Laboratories, Hercules, USA). Blots were then probed overnight at 4 °C or incubated for 1 h at room temperature with primary antibodies (anti-ß-Actin, anti-α-Tubulin, anti-PPAR-γ, anti-GAPDH, anti-UCP1, anti-ZIC1, anti-TBX1, and anti-24p3 receptor) diluted in LiCOR (Lincoln, NE, USA) blocking solution as per manufacturer’s recommendations. After incubation, blots were incubated with LiCOR secondary antibodies and visualized using the LiCOR Odyssey CXL imaging system software (Lincoln, NE, USA). For each protein of interest, the density value was normalized to the related density of the loading control to obtain the integrated density values. These values were then normalized to control samples run on the same gel.

### Statistical analysis

One-way ANOVA with Newmann-Keuls post-hoc test, Two-way ANOVA with Bonferroni post-Hoc test and non-parametric Two-tailed t-test (Mann-Witney) were performed to compare the statistical significance and represented as the mean ± SEM. P values < 0.05 were considered statistically significant. Statistical analysis and graphs were made using GraphPad Prism (version 6.07; La Jolla, CA, USA).

## Supplementary information

Supplementary Information
