# Supplementary material for: Lipocalin-2 deficiency may predispose to the progression of spontaneous age-related adiposity in mice
Source: Sci Rep. 2020 Sep 3;10:14589. doi: 10.1038/s41598-020-71249-7 (PMC7471318; doi:10.1038/s41598-020-71249-7)

Lcn2 deficiency may predispose to the progression of spontaneous age-related adiposity in mice

Keya Meyers, María López, Joanna Ho, Savannah Wills, Srujana Rayalam, Shashidharamurthy Taval

Supplementary Figure 1

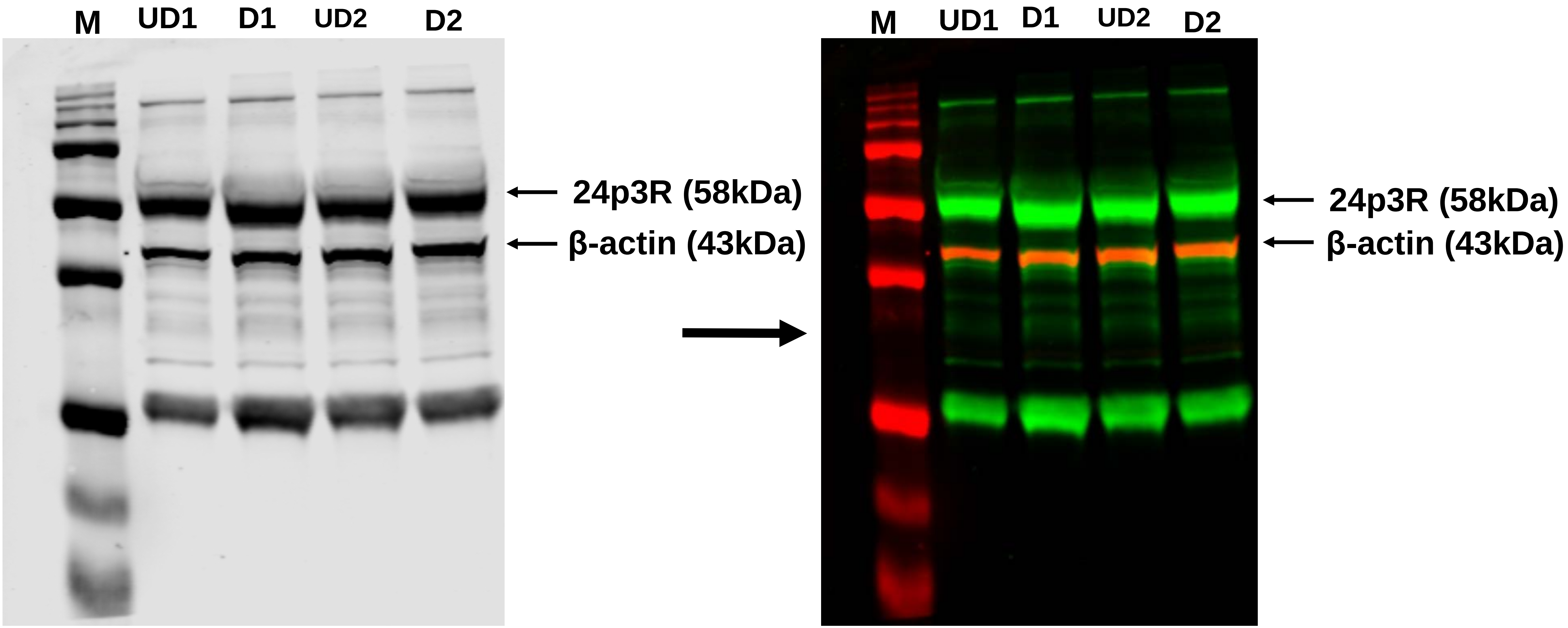

UD: Undifferentiated Adipocytes, D: Differentiated adipocytes.
Two individual experiments are ran on the same gel

Supplementary Figure 3A and B: Beigeing markers

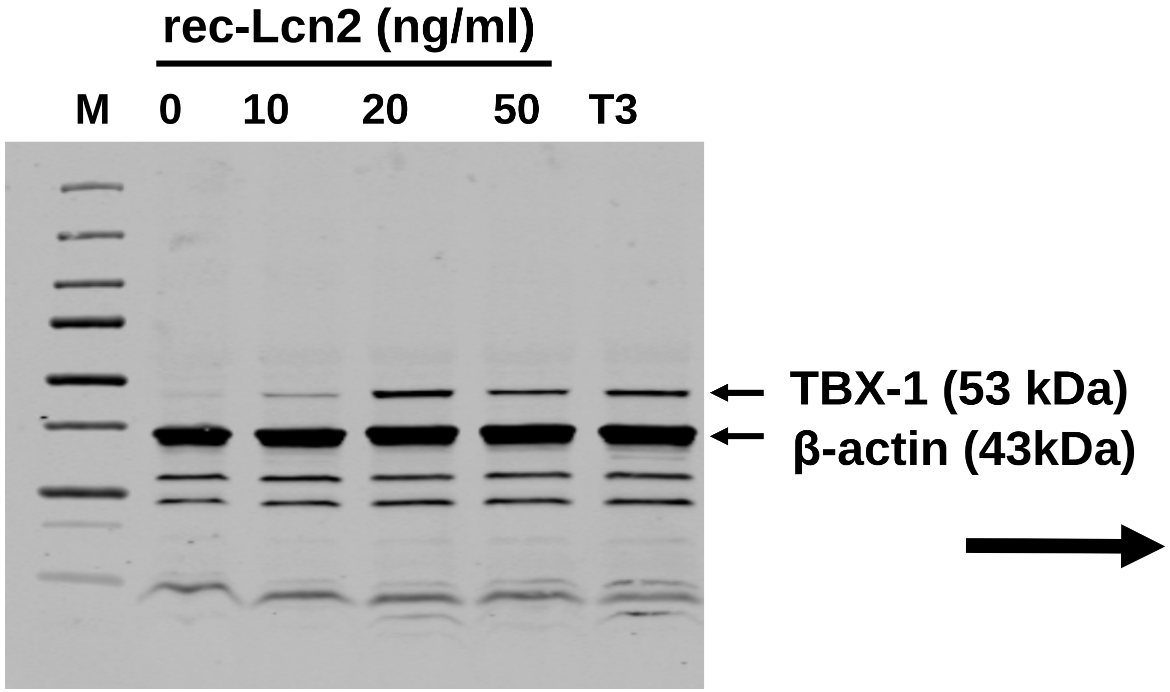

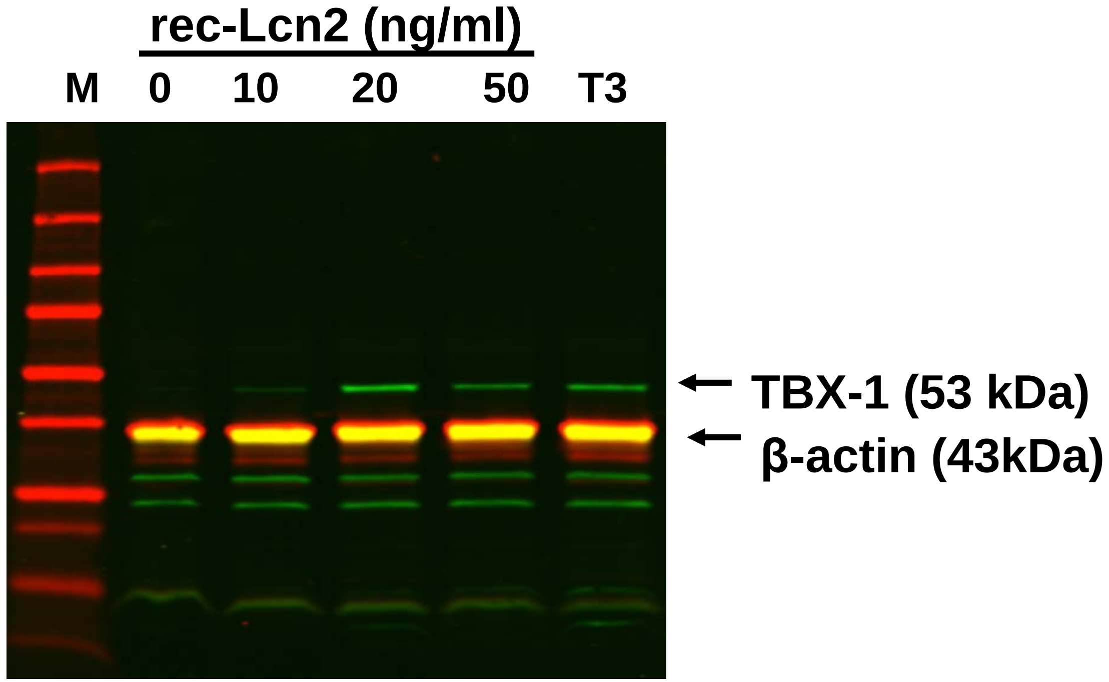

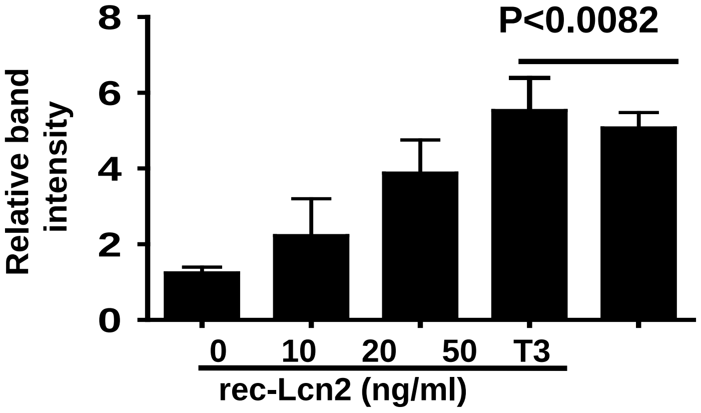

Supplementary Figure 3C and D: Beiging markers

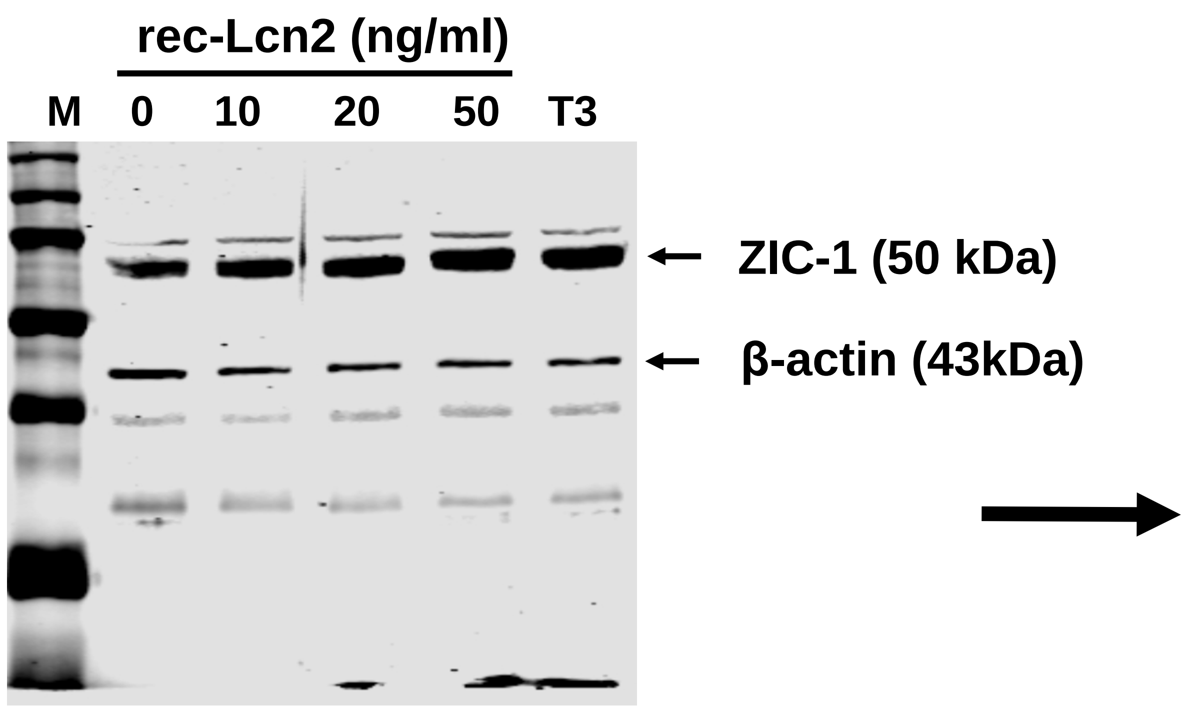

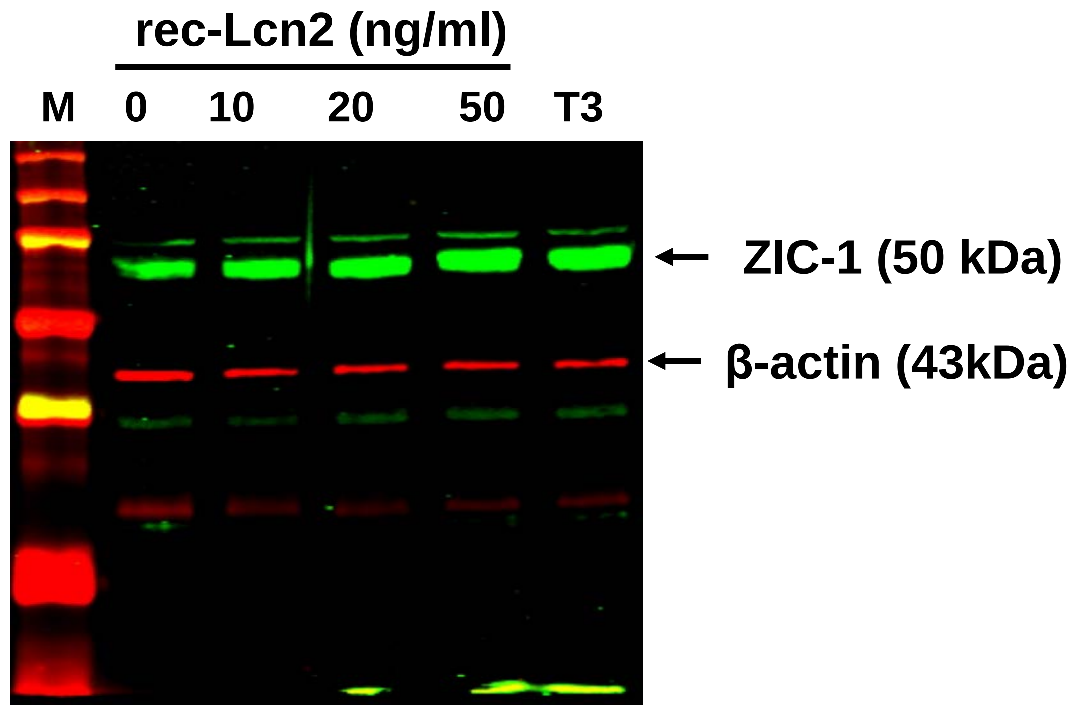

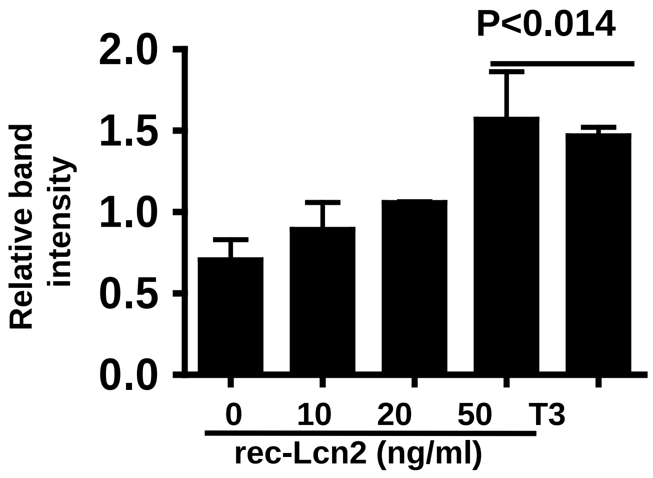

Supplementary Figure 3E and F: Thermogenic markers

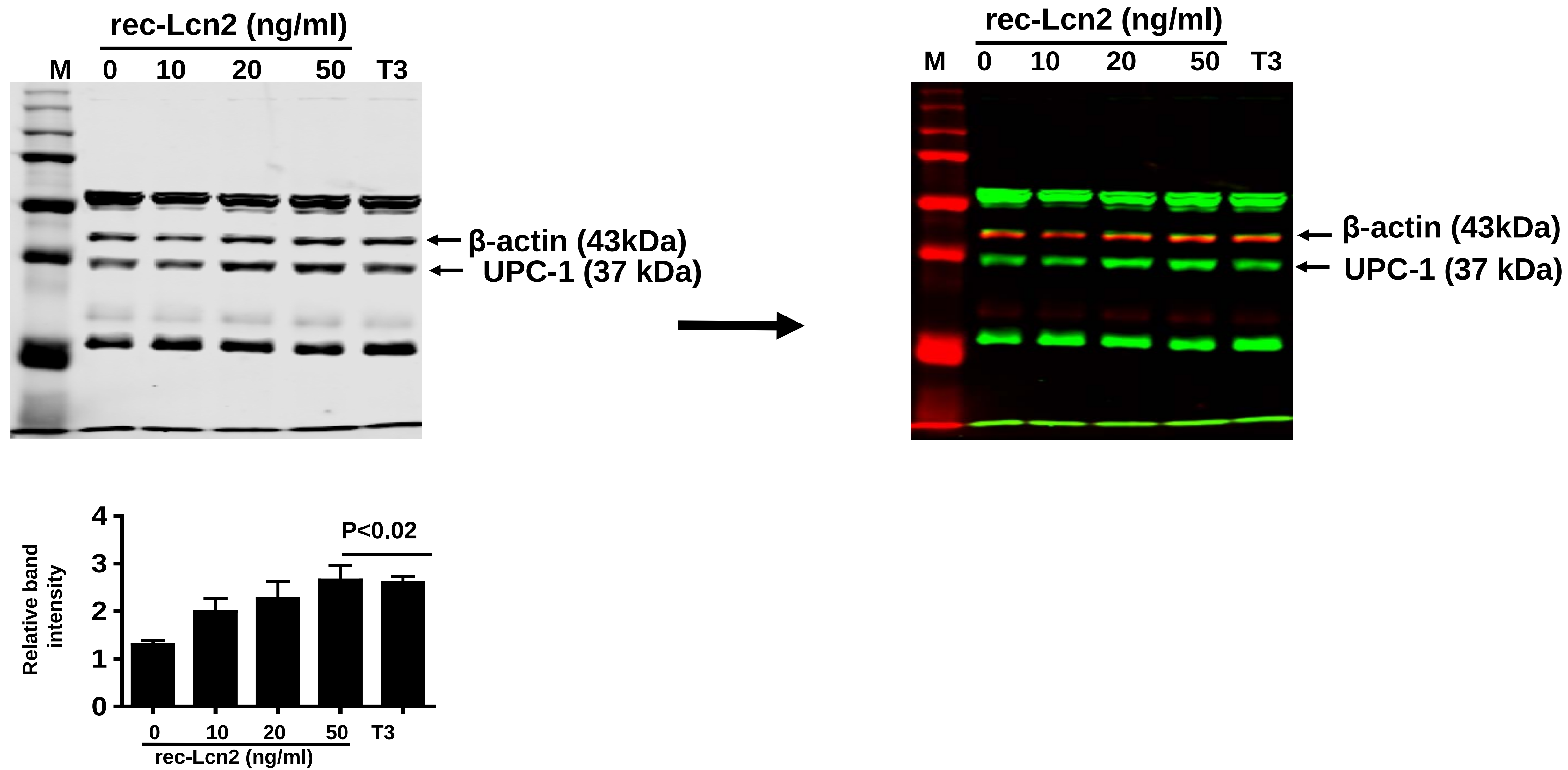

Supplementary Figure 3G and H: Thermogenic markers

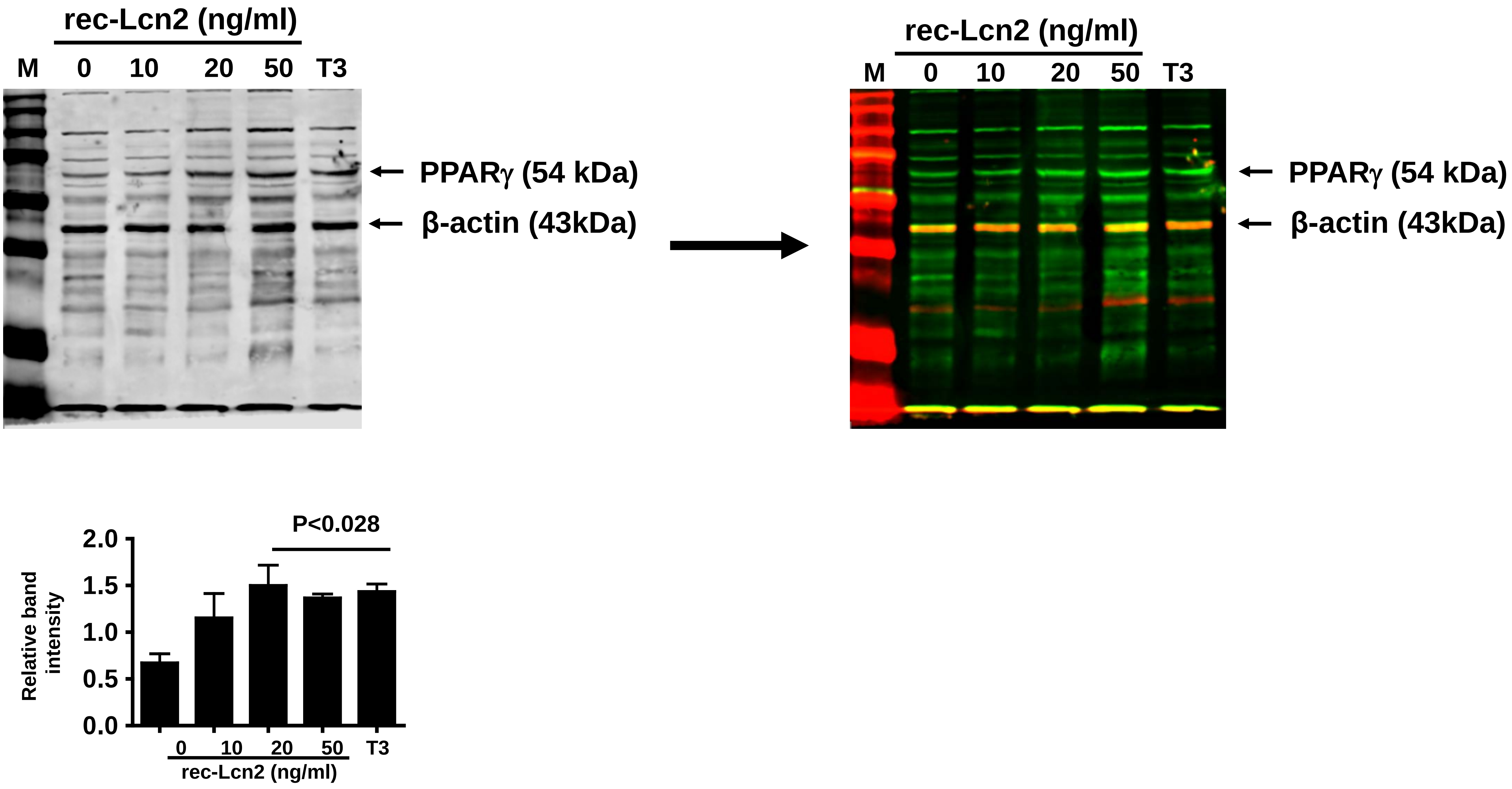

Supplement: Supplementary file 1 — Supplementary Information [file 41598_2020_71249_MOESM1_ESM.pdf]
